# Educational anxiety triggered by artificial intelligence technology: pathways and configuration analysis affecting teachers’ professional well-being

**DOI:** 10.3389/fpubh.2025.1717816

**Published:** 2026-01-08

**Authors:** Qing Wan, Hongmei Zhang, Jingjing Cao, Hui Tao

**Affiliations:** 1 UCL Institute of Education, University College London, London, United Kingdom; 2 International and Education Institute, Jiangxi Vocational College of Science and Technology, Nanchang, Jiangxi, China; 3 Shazhou Professional Institute of Technology, Zhangjiagang, China; 4 Faculty of Education, Languages and Psychology, SEGI University, Kuala Lumpur, Malaysia; 5 Academy for Educational Development and Innovation, The Education University of Hong Kong, Hong Kong, China

**Keywords:** artificial intelligence, educational anxiety, teachers’ professional well-being, self-determination theory, job crafting theory

## Abstract

**Introduction:**

This study aims to systematically explore the key factors influencing teachers’ professional well-being and to reveal the complex underlying mechanisms, with the goal of providing both theoretical and practical guidance for enhancing teachers’ professional well-being.

**Methods:**

A mixed-methods approach was adopted, combining Structural Equation Modeling (SEM) and Artificial Neural Networks (ANN) to analyze the influencing factors and pathways affecting teachers’ professional well-being.

**Results:**

First, work resources such as autonomy in instructional design, teacher-student relationships, sense of professional competence expansion, and proactive job crafting are significantly positively correlated with teachers’ professional well-being, while educational anxiety shows a significant negative correlation. Second, educational anxiety plays a crucial mediating role between work resources and well-being, indicating that part of the positive effect of these resources lies in alleviating anxiety. Third, work engagement positively moderates the beneficial effects of professional competence expansion and proactive job crafting on well-being.

**Discussion:**

The study suggests that administrators should grant teachers greater instructional autonomy and provide high-quality professional development opportunities to enhance their sense of competence. Schools should pay attention to teachers’ mental health by establishing support systems to alleviate educational anxiety. At the same time, guiding teachers to proactively engage in job crafting is an effective internal pathway to enhance individual well-being. This study offers a multidimensional theoretical perspective and practical reference for improving teacher well-being.

## 1 Introduction

Entering the third decade of the 21st century, artificial intelligence technology is permeating every aspect of the education sector at an unprecedented pace, driving profound changes in educational paradigms (1). From knowledge transmission to competency development, from standardized teaching to personalized learning, and from experience-driven to data-driven approaches, AI is redefining the essence and boundaries of education. In particular, the emergence of large language models represented by GPT-4, with their powerful capabilities in knowledge comprehension, reasoning, and creative writing, not only challenge traditional views of knowledge and learning but also fundamentally question the professional roles and value of teachers (2). This technology-driven educational transformation exhibits three notable characteristics: the acceleration of intelligent substitution—AI is rapidly taking over cognitive tasks that traditionally belonged to teachers (3); the blurring of functional boundaries—the role of teachers in human-machine collaborative teaching is becoming increasingly ambiguous (4); and the urgency of adaptation—teachers must quickly transition from being “knowledge authorities” to “learning designers.” Together, these features constitute the “technological dilemma” faced by contemporary teachers, namely, the deep-seated conflict between embracing technological innovation and maintaining professional dignity.

Teachers are the foundation and source of education; their professional well-being is not only an important indicator of individual quality of life but also directly affects the quality of teaching, student development, and the stability and progress of the entire educational system (5). In the current context of AI’s rapid integration into education, neglecting the negative psychological impacts of technological change on teachers may lead to increased professional burnout, loss of outstanding talent, and a series of related issues, thereby undermining the original intention of empowering education through technology (6). However, existing research on the integration of AI and education generally falls into two mainstream paradigms. The first is the “technology-centric” approach, which focuses on the design, development, and application of AI technologies, exploring how to optimize algorithms and build platforms to better “empower” teaching, with an emphasis on technology and application (7). The second is the “macro-ethical critique” approach, which examines the potential macro-level risks of AI in education—such as data privacy, algorithmic bias, educational equity, and the loss of human subjectivity—from philosophical, sociological, and ethical perspectives, focusing on ethics and norms (8).

Although these studies provide important technical blueprints and ethical frameworks for understanding AI in education, they commonly overlook a crucial intermediate link: the internal “psychological perceptions and stress responses” of teachers as the main agents of technology adoption and practice. Technology does not directly and objectively impact educational outcomes; rather, it must pass through the “black box” of teachers’ cognition, interpretation, emotional experience, and behavioral choices to be transformed into real educational practice. When AI technology enters the professional domain of teachers in a disruptive manner, it triggers not only operational issues at the application level but also profound psychological shocks related to professional identity, self-worth, and future expectations. Therefore, the academic community urgently needs to shift its research perspective from external “technological empowerment” or “ethical scrutiny” to the individual “psychological perception” of teachers, exploring the ripple effects of technological change at the micro-psychological level.

At the practical level, while artificial intelligence technology brings opportunities, it also gives rise to a pervasive form of occupational anxiety—“AI education anxiety.” This anxiety is not unfounded; rather, it is rooted in multiple real-world contradictions. First is the sense of threat from “role replacement,” as AI’s high efficiency in traditional teaching tasks such as knowledge transmission and grading has caused some teachers to question their core value (9). Second is the pressure of a “skills deficit,” with rapid technological iteration requiring teachers to continuously acquire new skills, creating immense pressure to transition from “digital immigrants” to “AI natives (10).” Third is the confusion surrounding “human-machine collaboration,” as there is a lack of mature theoretical guidance and practical models for how teachers can complement AI in teaching rather than simply being replaced, and for how to guide students in the critical use of AI (11). Fourth is the sense of powerlessness from “imbalanced evaluation,” as the current evaluation system still focuses on traditional knowledge assessment, making it difficult for teachers to gain recognition for their efforts in exploring innovative AI-integrated teaching, which in turn leads to professional burnout (12).

This complex anxiety has become a significant source eroding teachers’ professional well-being. Professional well-being refers to teachers’ overall, positive emotional experience and value judgment regarding their professional careers, and serves as a key psychological capital for maintaining the vitality and creativity of the educational system (13). Numerous studies have confirmed that sustained occupational stress and anxiety are core factors leading to decreased professional well-being and increased turnover intentions among teachers (14). However, is the relationship between AI education anxiety and professional well-being simply a linear negative correlation, or is there a more complex mediating process? This is a key question that urgently needs to be addressed.

Therefore, this study focuses on the core construct of “AI education anxiety,” aiming to reveal its internal mechanisms affecting teachers’ professional well-being. The innovations of this paper are as follows: First, it breaks away from the binary perspective of “technological empowerment” or “ethical risk” in previous research on AI, and instead approaches the issue from the perspective of teachers’ “psychological perception,” taking “anxiety” as the core concept to explore the internal psychological processes through which technological change acts on individuals, thus aligning more closely with the realities of educational practice. Second, it systematically incorporates AI-induced professional achievement, teachers’ professional well-being, and multidimensional moderating/protective factors into a unified analytical framework, enabling an in-depth exploration of the issue from “what it is” to “why” and “how.” Third, it employs structural equation modeling (SEM) to test the hypothesized model constructed based on existing theories, clearly revealing the linear and directional transmission chains among variables. On this basis, an artificial neural network (ANN) model is introduced, with all antecedent variables as the input layer and teachers’ professional well-being as the output layer, to construct a high-precision predictive model.

To achieve the above research objectives, this study will focus on the following core questions: (1) In the context of the increasing prevalence of AI technology, what are the overall conditions of AI education anxiety and professional well-being among Chinese teachers? Is there a significant correlation between the two? (2) Through which core psychological pathways does AI education anxiety affect teachers’ professional well-being, and what is the strength of these effects? Clarifying these issues is of great theoretical and practical significance for building a new ecology of future education based on human-machine collaboration, formulating scientific teacher support policies, and enhancing teachers’ adaptability and professional well-being.

## 2 Literature review and theoretical foundation

### 2.1 The reshaping and challenges of the teacher’s role by artificial intelligence

Artificial intelligence technology, especially the rise of generative AI, is triggering a profound transformation in educational paradigms, with its core lying in the fundamental reshaping of the traditional teacher’s role (15). Firstly, AI, as a “teaching efficiency enhancer,” can take over a large number of repetitive and transactional tasks, such as grading assignments, analyzing learning data, and answering basic queries, thereby freeing teachers from burdensome administrative and basic assessment duties (16). This trend toward automation is not intended to replace teachers, but rather to shift their focus from being “transmitters of knowledge” to more advanced roles.

This evolution is reflected in the diversification and upgrading of the teacher’s role. On the one hand, teachers are increasingly acting as “learning designers” and “guides in the growth process.” They need to utilize personalized learning data provided by AI to design differentiated learning paths for students and offer more targeted emotional support and higher-order thinking guidance (17). On the other hand, as human-machine collaboration becomes the new norm in teaching, the teacher’s role is elevated to that of a “classroom conductor.” Teachers must dynamically coordinate students, AI teaching assistants, and diverse digital resources, making real-time decisions in complex teaching environments to achieve complementary advantages between humans and machines and maximize learning outcomes (18). In this model, the unique value of teachers is no longer the monopoly of knowledge, but rather their judgment in complex situations, empathy, and care for the holistic development of students.

However, this profound reshaping of the teacher’s role does not happen overnight; it is accompanied by a series of severe practical challenges, which constitute the direct source of AI education anxiety. First is the daunting “capability gap” challenge. Teachers not only need to master the technical literacy to operate AI tools but also need to develop “new pedagogies for the AI era,” such as how to guide students in critical human-machine interaction and how to assess the quality of AI-generated content. This places enormous pressure on existing teacher professional development systems (19). Second is the deep “professional identity” crisis. As AI becomes capable of handling more and more traditional teaching tasks, some teachers may experience a sense of “deskilling,” leading to doubts about their core value and professional irreplaceability, and thus triggering role ambiguity and existential anxiety (20). Third is the sharply increased “cognitive load” pressure. While the role of “classroom conductor” sounds creative, it also requires teachers to monitor students’ learning status while managing complex interactions with AI systems, imposing unprecedented demands on their cognitive resources (21). In summary, while AI endows teachers with new potential, it also places them at a crossroads full of uncertainty and challenges. The stress and confusion arising from this role reshaping are precisely the core factors of concern in this study.

### 2.2 Educational anxiety under the impact of artificial intelligence

The rapid development of artificial intelligence technology and its outstanding performance in cognitive tasks are triggering profound educational anxiety worldwide (22). This anxiety is defined in the literature as a systemic concern over the declining value of future skills, resulting from the “dual impact” of AI on both the labor market and traditional educational models. Firstly, the core driving force of educational anxiety is the “perceived obsolescence of skills” caused by AI’s impact on the job market. Studies indicate that the widespread adoption of generative AI is automating routine tasks of knowledge workers at an unprecedented pace, leading students and families to seriously question the future value of current educational investments (23). This uncertainty compels the education system to undergo an ontological shift, redirecting its focus from knowledge transmission to the cultivation of higher-order cognitive and emotional intelligence skills that are difficult for AI to replace, such as creative thinking, ethical judgment in complex situations, and cross-cultural collaboration abilities (24).

Secondly, teachers are also required to rapidly integrate AI tools (such as intelligent tutoring systems and content generators) to meet the new demands of personalized learning and data-driven instruction. However, this integration often occurs without adequate training and technical support, resulting in significant “technological stress” for teachers (25). Research shows that when teachers perceive themselves as lacking technological competence or are faced with the substantial cognitive load brought by AI systems, their sense of teaching self-efficacy drops sharply, which is a key negative predictor of professional well-being (22). In addition, educational anxiety compels educational administrations to rely on AI for more refined performance evaluations and instructional monitoring. Teachers begin to worry that their professional judgment and classroom interactions are being excessively managed or replaced by algorithms (26). This fear of “algorithmic management” calls into question the teacher’s role as an educational, emotional, and ethical guide. When teachers feel that their work is being reduced to tasks that can be replicated or supervised by machines, their intrinsic motivation and sense of professional engagement rapidly decline, directly eroding the psychological foundation of professional well-being (27).

In summary, the academic community currently regards educational anxiety under the impact of artificial intelligence as a complex socio-technical phenomenon. It concerns not only the substitution of skills by technology, but also the redefinition of educational equity, teachers’ mental health, and social adaptability. The key to alleviating this anxiety lies in innovative educational policies that ensure the education system can rapidly and equitably develop “uniquely human skills,” thereby buffering the systemic pressures on teachers and rebuilding their professional value and well-being in an AI-empowered environment.

### 2.3 Teacher professional well-being

Teacher professional well-being is a core research topic in educational psychology and organizational behavior, widely defined as a multidimensional construct that goes beyond mere salary satisfaction, encompassing the positive psychological and emotional states, work engagement, and sense of meaning experienced by teachers throughout their careers (28). Research generally holds that professional well-being is not only an important indicator of teachers’ mental health, but also a key predictor of educational quality, teacher retention rates, and student academic achievement (29). Theoretically, self-determination theory (SDT) provides a solid foundation for understanding the internal mechanisms of teacher professional well-being. Studies indicate that teachers’ well-being is closely related to the fulfillment of three basic psychological needs: autonomy, meaning teachers have the right to make choices and decisions in their teaching practice; competence, referring to teachers’ perceived teaching self-efficacy and professional ability; and relatedness, which involves establishing positive social relationships with colleagues, students, and administrators (30). When these needs are met, teachers experience higher work engagement, intrinsic motivation, and emotional stability, thereby achieving a state of “professional flourishing (31).”

In summary, the academic community currently regards teacher professional well-being as a multidimensional and dynamically changing psychological and social phenomenon. It not only concerns teachers’ personal professional identity and sense of achievement, but also profoundly influences educational quality, teacher-student relationships, and the overall school climate. The enhancement of teacher professional well-being depends both on external factors such as policy support, compensation, and social respect, and on teachers own professional development and psychological adjustment abilities. The key to achieving teacher’ professional well-being lies in building a highly supportive and developmental educational ecosystem. By optimizing management mechanisms, improving incentive measures, and strengthening mental health services, teachers can realize their self-worth in an ever-changing educational environment, enhance their sense of professional belonging, and thus promote the stability of the teaching workforce and the sustainable development of education.

### 2.4 Self-determination theory

SDT, established and continuously developed by Deci and Ryan, is a macro-level motivation theory whose core idea is that all human beings possess three intrinsic and universal psychological needs: autonomy, competence, and relatedness (32). The satisfaction of these needs forms the foundation for optimal functioning, psychological growth, and well-being (33). In the field of education, SDT has become a powerful theoretical framework for explaining teachers’ work motivation, occupational burnout, and well-being (30). Traditional research has confirmed that when teachers experience teaching autonomy (autonomy), believe in their ability to accomplish teaching tasks (competence), and establish supportive relationships with colleagues and students (relatedness), their professional well-being and work engagement are significantly higher, while turnover intention and occupational burnout are notably lower (34).

The need for autonomy refers to the individual’s sense that their actions are self-endorsed and aligned with their values. However, the introduction of AI technology may undermine teachers’ professional autonomy. For example, standardized AI teaching platforms and adaptive learning systems may predefine instructional paths and content, shifting teachers’ roles from curriculum designers to executors and supervisors of AI systems (20). Some studies have pointed out that AI-based instructional assessment and monitoring systems may trigger a sense of “being surveilled” among teachers, making their teaching behaviors more conservative and compliant, and further constraining their space for professional autonomy and innovation (35). This aligns precisely with SDT’s view that “controlled motivation” leads to decreased well-being.

The need for competence refers to the individual’s perception of being able to meet environmental challenges and achieve expected outcomes. AI technology is reshaping the core competencies required in education at an unprecedented pace, bringing about a significant “competence shock” for teachers. Teachers may experience anxiety over “skill obsolescence,” worrying that they cannot master complex new technologies or that their traditional teaching skills pale in comparison to AI (36). When teachers perceive a substantial gap between their digital literacy and the demands of AI application, they may experience strong feelings of inadequacy and self-doubt, directly threatening the satisfaction of their competence needs (37). This “competence panic” triggered by technological generational shifts is a deeper psychological threat than routine work challenges, as it shakes the very foundation of teachers’ professional identity.

The need for relatedness refers to the individual’s desire to establish secure and caring connections with others. Education is essentially an interpersonal endeavor, and harmonious relationships with students and colleagues are important sources of teacher well-being (38). Excessive intervention by AI may lead to the “dehumanization” of the educational process. For example, when AI chatbots take on most of the Q&A and emotional support functions, meaningful and spontaneous interactions between teachers and students may decrease (39). Meanwhile, within schools, varying levels of AI application may create new “digital divides” and competitive pressures, undermining the original collaborative and supportive relationships among teachers and causing some to feel isolated. This relational alienation brought about by “technological mediation” directly undermines teachers’ need for relatedness.

### 2.5 Job crafting theory

Job crafting theory was first proposed by Wrzesniewski and Dutton (40). Its core idea is that employees proactively and bottom-up adjust the task boundaries, relational boundaries, and cognitive boundaries of their work to make it better align with their personal values, strengths, and passions. This proactive behavior is not about waiting for the organization to redesign work from the top down, but rather an adaptive strategy individuals adopt to enhance the meaning, engagement, and sense of efficacy in their work. Traditional job crafting includes three dimensions: task crafting—changing the form, scope, or amount of work tasks; relational crafting—altering the targets, frequency, or quality of interpersonal interactions at work; and cognitive crafting—changing the perception and interpretation of the purpose and meaning of work (41).

Existing research shows that in the face of AI technology’s penetration, teachers’ job crafting behaviors are mainly reflected in three aspects: task crafting, relational crafting, and cognitive crafting (42). First, task crafting is manifested in teachers’ proactive learning of AI-related skills and integrating AI tools into instructional design and classroom management, thereby enhancing their technological adaptability and teaching innovation (43). For example, some teachers expand their professional boundaries and alleviate career anxiety caused by technological substitution by participating in new tasks such as AI curriculum development and data analysis (44). Second, relational crafting is reflected in teachers establishing new collaborative networks with technical experts, colleagues, and students to jointly explore AI-empowered teaching models. Such interdisciplinary and cross-role collaboration not only facilitates knowledge sharing and capacity building but also enhances teachers’ sense of belonging and professional identity (45). Finally, cognitive crafting refers to teachers’ re-cognition of the relationship between AI technology and education. Some teachers redefine their value in the intelligent education environment, viewing AI as an enabler rather than a threat, thus transforming anxiety into motivation for growth (46). This positive cognitive crafting helps teachers maintain psychological resilience and professional enthusiasm amid technological change.

In summary, job crafting theory not only reveals teachers’ proactive adaptation mechanisms under the impact of AI technology but also provides practical pathways for enhancing teacher well-being and alleviating educational anxiety. Future research could further explore the interactions among different types of job crafting behaviors and their long-term effects on teacher well-being, especially in the ever-evolving educational ecosystem shaped by AI technology.

### 2.6 Research hypotheses

Against the backdrop of artificial intelligence technology deeply empowering education, the mechanisms influencing teachers’ professional well-being have become increasingly complex. To further explore how AI technology affects teachers’ professional well-being by fulfilling their basic psychological needs, this study adopts SDT and Job Crafting Theory as its core theoretical perspectives to construct a systematic research framework, as shown in Table 1. AI technology not only provides teachers with greater autonomy in instructional design (TDA), a sense of professional competence expansion (TSC), and enhanced teacher-student relationship connectedness (PCE)—thus meeting their three basic psychological needs of autonomy, competence, and relatedness—but also stimulates teachers’ initiative in job crafting (PJC), reflecting a shift from passive adaptation to proactive creation, as illustrated in Table 1.

**Table 1:** Correspondence between variables related to teacher professional well-being and theoretical foundations.

Variable	Theory	
Teaching design autonomy (TDA)	Meet independent needs	Self-determination theory
Teacher-student connection (TSC)	Satisfy belonging needs	
Professional competence expansion (PCE)	Meet capacity needs	
Proactive job crafting (PJC)	Teachers’ initiative from passive acceptance to active creation	Job crafting theory

**Figure 1:**
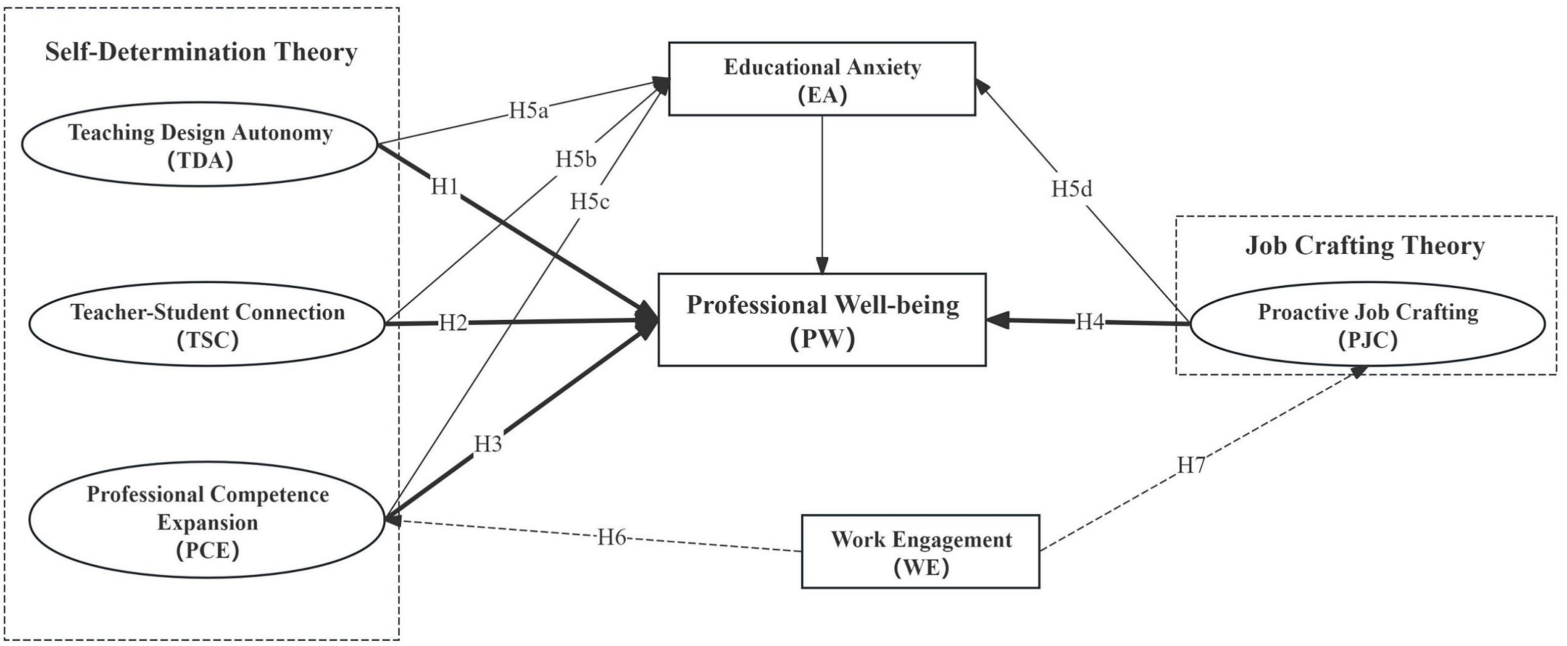
Hypothetical framework diagram.

Based on the above theoretical analysis, this study further focuses on the moderating role of teacher work engagement in the aforementioned relationships, as well as the mediating role of educational anxiety in the process by which AI technology influences teacher professional well-being. Specifically, the level of teacher work engagement in an AI environment may strengthen or weaken the positive impact of AI technology on their professional well-being; meanwhile, educational anxiety may serve as a mediating variable between AI technology and professional well-being, affecting teachers’ psychological experiences and behavioral responses. The hypothesized model of this study is shown in Figure 1.

#### 2.6.1 The impact of self-determination theory on teacher professional well-being

SDT posits that when individuals’ needs for autonomy, competence, and relatedness are satisfied, their intrinsic motivation is stimulated, thereby enhancing their well-being. In the context of AI-empowered education, technological empowerment is expected to fulfill these three basic psychological needs of teachers. First, AI tools liberate teachers from repetitive tasks, granting them more time and energy for creative instructional design, thus satisfying their need for autonomy. Second, AI-assisted precision teaching provides teachers with more opportunities for high-quality teacher-student interactions and emotional support, deepening teacher-student relationships and fulfilling the need for relatedness. Finally, data insights and cutting-edge knowledge provided by AI can significantly enhance teachers’ instructional decision-making abilities and professional vision, meeting their need for competence.

Accordingly, this study proposes the following hypotheses:

*H1*: Teaching design autonomy has a significant positive effect on teacher professional well-being.

*H2*: Teacher-student connection has a significant positive effect on teacher professional well-being.

*H3*: Professional competence expansion has a significant positive effect on teacher professional well-being.

#### 2.6.2 The impact of job crafting theory on teacher professional well-being

Job Crafting Theory posits that employees are not passive recipients of work arrangements, but rather proactively adjust their work tasks, interpersonal relationships, and professional cognition to make their work more meaningful and satisfying. In the face of the challenges and opportunities brought by AI technology, teachers’ proactivity is particularly crucial. Proactive teachers engage in task crafting (such as optimizing work processes with AI), relational crafting (such as using AI data to better interact with students and parents), and cognitive crafting (such as redefining their roles as “learning designers” and “growth companions”). This proactive job crafting enables them to better adapt to the new technological environment and derive professional satisfaction from it.

Accordingly, this study proposes the following hypotheses:

*H4*: Teachers’ proactive job crafting has a significant positive effect on professional well-being.

*H4a*: Task crafting has a significant positive effect on teacher professional well-being.

*H4b*: Relational crafting has a significant positive effect on teacher professional well-being.

*H4c*: Cognitive crafting has a significant positive effect on teacher professional well-being.

#### 2.6.3 The mediating role of educational anxiety

Although AI technology brings opportunities, it is also accompanied by challenges, giving rise to teacher-specific educational anxieties, such as anxiety over the application of new technologies, concerns about professional replacement and self-worth, and anxiety regarding the uncertainty of teaching outcomes. These anxieties constitute key psychological pathways affecting teachers’ professional well-being. On the one hand, the challenges and threats brought by technology directly generate feelings of anxiety; on the other hand, such anxiety depletes teachers’ psychological resources, reduces their job satisfaction and sense of achievement, and thus undermines their professional well-being. Therefore, educational anxiety may play a mediating role between AI contextual factors and teachers’ professional well-being. Accordingly, this study proposes the following hypotheses:

*H5a*: Educational anxiety mediates the relationship between teaching design autonomy and teacher professional well-being.

*H5b*: Educational anxiety mediates the relationship between teacher-student connection and teacher professional well-being.

*H5c*: Educational anxiety mediates the relationship between perceived professional competence expansion and teacher professional well-being.

*H5d*: Educational anxiety mediates the relationship between proactive job crafting and teacher professional well-being.

#### 2.6.4 The moderating role of teacher work engagement

Against the backdrop of artificial intelligence technology continuously permeating the field of education, teachers’ perceived professional competence expansion and proactive job crafting have become important factors influencing their professional well-being. However, when facing new technologies and tasks, teachers’ subjective experiences and behavioral responses are not entirely consistent. Existing research indicates that individual work engagement plays a key moderating role between changes in the external environment and professional well-being. Work engagement refers to the positive psychological state exhibited by individuals at work, mainly including vigor (i.e., the energy and resilience teachers display in their work), dedication (i.e., teachers’ enthusiasm for and sense of meaning in their work), and absorption (i.e., the state of being fully immersed in work). A high level of work engagement not only helps teachers better adapt to changes brought by AI technology but also enhances their positive experiences when facing challenges, thereby improving their professional well-being.

Specifically, when teachers experience an expansion of professional competence empowered by AI technology, a high level of work engagement enables them to more actively transform new competencies into a sense of professional achievement and psychological satisfaction. Similarly, when teachers proactively engage in job crafting, a high level of work engagement helps them better cope with changes and realize self-value. Therefore, work engagement may play a moderating role between perceived professional competence expansion, proactive job crafting, and teacher professional well-being. Based on this, the following research hypotheses are proposed:

*H6*: Work engagement moderates the relationship between perceived professional competence expansion and teacher professional well-being.

*H7*: Work engagement moderates the relationship between proactive job crafting and teacher professional well-being.

## 3 Research methods and model construction

### 3.1 Research methods

To systematically explore the impact pathways and configuration mechanisms of educational anxiety induced by artificial intelligence technology on teachers’ professional well-being, this study adopts a mixed-methods approach combining SEM and artificial neural networks (ANNs). The specific research methods and procedures are as follows:

First, based on a preliminary literature review and theoretical analysis, the research variables and their secondary dimensions were identified. A questionnaire was designed to include variables such as autonomy in instructional design, depth of teacher-student relationship, perceived professional competence expansion, proactive job crafting, work engagement, educational anxiety, and teacher professional well-being. The questionnaire was measured using a five-point Likert scale and was revised and refined through expert review and a small-scale pilot survey. Second, a stratified random sampling method was used to select teachers from different regions and educational stages as survey participants, and valid questionnaires were distributed and collected. The collected data underwent preprocessing, including handling missing values, eliminating outliers, and conducting reliability and validity tests to ensure data quality.

**Figure 2:**
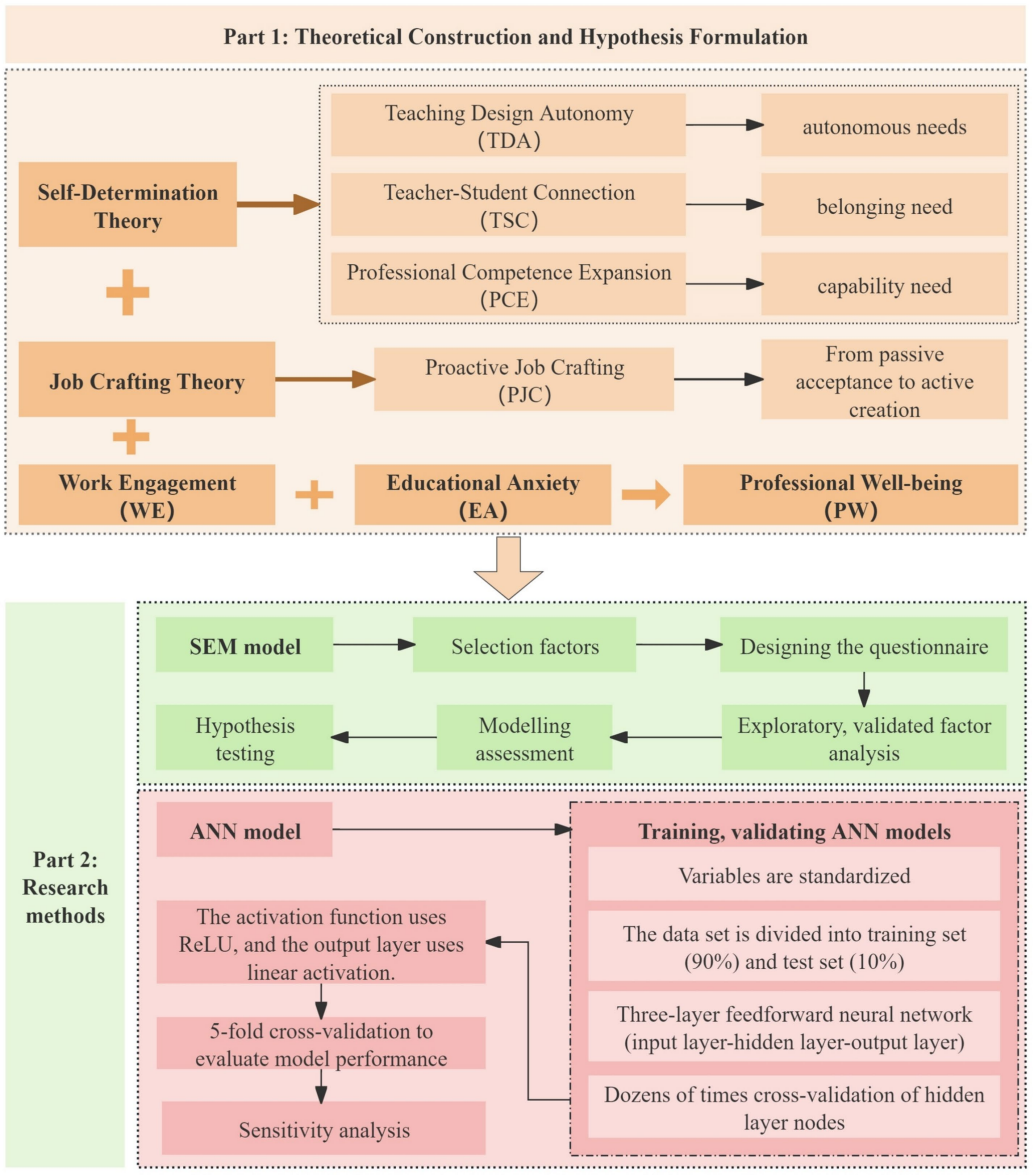
Research flowchart.

In the data analysis stage, SEM was first used to verify the relationships among variables. SEM can simultaneously handle complex relationships among multiple variables, test the model fit, and analyze both direct and indirect effects of each path (47). Through SEM analysis, the impact pathways and significance of various factors on teachers’ professional well-being can be clarified. On this basis, the ANN model was further introduced to supplement and optimize the SEM analysis results. As a nonlinear modeling tool, ANN can capture complex nonlinear relationships among variables, enhancing the model’s predictive power and explanatory capacity (48). The ANN model can identify key variable combinations and their relative importance in influencing teachers’ professional well-being, thus providing data support for subsequent pathway and configuration analysis. Finally, combining the results of SEM and ANN analyses, this study comprehensively discusses the mechanisms by which variables such as educational anxiety and work engagement affect teachers’ professional well-being in the context of AI technology, and proposes targeted policy recommendations and practical implications. To strictly adhere to the principles of transparency and reproducibility in scientific research, the analytical procedure and parameter settings of the SEM-ANN approach in this study are illustrated in Figure 2.

### 3.2 Variable design

This study collected data through a structured questionnaire survey, with the questionnaire content closely centered on core variables such as educational anxiety and teachers’ occupational well-being under the influence of artificial intelligence technology. The questionnaire was divided into two main sections: (1) Respondents’ demographic characteristics, including basic information such as gender, age, years of teaching experience, educational background, teaching stage, and school type, in order to describe the sample structure and facilitate subsequent subgroup analyses; (2) Measurement items for core variables, covering primary dimensions and their secondary explanatory variables such as autonomy in instructional design, sense of connection in teacher-student relationships, perceived expansion of professional competence, proactive job crafting, work engagement, educational anxiety, and teachers’ occupational well-being. All measurement items for independent variables were adapted from well-established domestic and international scales. Specifically, autonomy in instructional design, sense of connection in teacher-student relationships, and perceived expansion of professional competence were derived from SDT, while proactive job crafting was based on Job Crafting Theory, with appropriate adjustments made to reflect the practical application of artificial intelligence technology in education. In total, 12 measurement items were included. All items were rated on a five-point Likert scale (1 = “strongly disagree,” 5 = “strongly agree”), with specific measurement content shown in Table 2.

**Table 2:** Questionnaire design.

First-level dimension	Second-level explanatory variable	Questionnaire question
Teaching design autonomy (TDA)	Creative empowerment	AI tools can provide abundant teaching inspiration and materials, making it easier to design creative lessons.
	Personalized teaching implementation	With the help of AI, it is easier to design differentiated learning paths for students of different levels, achieving personalized teaching.
	Time resource reallocation	AI can free me from repetitive lesson preparation tasks, giving me more time to consider higher-level teaching strategies.
Teacher-student connection (TSC)	Data-driven decision-making ability	Using AI teaching analytics tools enables more accurate teaching decisions based on data.
	Access to cutting-edge knowledge	Through interaction with AI, I can quickly access and understand the latest developments in my subject, expanding my knowledge boundaries.
	Interdisciplinary teaching ability	AI technology makes it easier to integrate knowledge or methods from other disciplines into my teaching.
Professional competence expansion (PCE)	Precision care implementation	AI’s precise early warning of students’ learning difficulties allows me to provide more timely and targeted care and guidance.
	Increased high-quality interaction time	Because AI handles a large amount of administrative and grading work, I have more time for in-depth one-on-one communication with students.
	Expansion of interaction topics	AI tools have become new and interesting topics of interaction and areas for joint exploration between teachers and students.
Proactive job crafting (PJC)	Task crafting	I consciously seek out and use AI tools to automate work tasks I dislike (such as repetitive grading).
	Relationship crafting	I actively use AI as a medium to create new ways of collaboration and interaction with students or colleagues.
	Cognitive crafting	I tend to view the challenges brought by AI as opportunities to improve myself and make my work more interesting.
Work engagement (WE)	Vigor	Refers to the high level of energy and psychological resilience an individual demonstrates at work.
	Dedication	Refers to the enthusiasm, sense of meaning, and pride an individual feels toward their work.
	Absorption	Refers to being fully immersed in work, to the extent that it is difficult to disengage.
Educational anxiety (EA)	Technology application anxiety	Teachers worry that due to a lack of knowledge and skills in using AI technology, they may not be able to master or effectively apply these new technologies.
	Job replacement anxiety	Teachers are concerned that AI technology will gradually replace some or even core teaching functions.
	Teaching effectiveness anxiety	Worry that AI technology may affect the effectiveness of traditional teaching or their own teaching abilities.
Professional well-being (PW)	Sense of Achievement	The sense of accomplishment teachers gain from their work.
	Professional identity	Teachers’ sense of identification and belonging to their profession.
	Mental health	Teachers’ mental health status and stress levels.

To ensure the scientific rigor and validity of the questionnaire, the initial draft was reviewed by several experts in education, psychology, and artificial intelligence to guarantee the professionalism and applicability of the content. Subsequently, a small-scale pilot test was conducted, and some expressions were optimized and revised based on feedback from participating teachers. The final version of the questionnaire was well-structured and scientifically sound, comprehensively reflecting the actual status of core variables such as teachers’ educational anxiety, work engagement, and occupational well-being in the context of artificial intelligence application. This provided a solid data foundation for subsequent empirical analysis and model testing.

### 3.3 Data collection

This study targeted in-service teachers within China’s K-12 education system (including primary, junior high, and high schools) as research participants, and collected first-hand data through a questionnaire survey. All participants were adults, and the study received approval from the Ethics Committee of Shazhou Professional Institute of Technology prior to implementation. Informed consent was obtained from all participants before they completed the questionnaire; participants were clearly informed of the study’s purpose, content, data usage, and their right to voluntary participation, with the option to withdraw at any stage. To protect participants’ privacy, no personally identifiable information was collected in the questionnaire. All data were anonymized and securely stored in an encrypted database, accessible only to members of the research team.

This study was conducted exclusively in mainland China and did not involve Malaysia. To ensure the representativeness and diversity of the sample, the research team employed a stratified random sampling method, stratifying by geographic region (eastern, central, and western China), school type (primary, junior high, and high school), and urban–rural distribution. The sample covered six provinces and municipalities, including Beijing, Shanghai, Jiangsu, Guangdong, Sichuan, and Shaanxi, encompassing first-tier cities, second-tier cities, and rural areas. Participating teachers were required to meet the following criteria: (1) currently teaching in a K-12 school in mainland China; (2) holding a teaching qualification certificate; (3) having at least 1 year of teaching experience; and (4) voluntarily participating in this study.

The questionnaire was distributed from February to June 2025, using a combination of online and offline methods. Online distribution mainly relied on major educational forums, professional teacher communities, and social media platforms; offline distribution was conducted in selected partner schools, where teachers completed the questionnaire collectively after obtaining consent from both the schools and the teachers. A total of 450 questionnaires were distributed. After rigorous screening of the returned questionnaires—excluding those with excessively short completion times, obvious response patterns, or logical inconsistencies—a total of 363 valid questionnaires were obtained, resulting in a valid response rate of 80.67%. The educational backgrounds of the participating teachers ranged from associate degrees and bachelor’s degrees to master’s degrees and above, and their subject backgrounds included Chinese, mathematics, English, science, arts, and other disciplines. The high quality of the valid samples provided a solid foundation for the accuracy and reliability of subsequent data analysis.

### 3.4 Sample characteristics

To clearly present the sample composition, descriptive statistical analysis was conducted on the demographic characteristics of the 363 valid samples. In terms of demographic characteristics, the teacher sample in this survey is highly representative. Specifically, in terms of gender distribution, female teachers accounted for the majority (71.1%), which aligns with the general gender structure of the teaching workforce in China. Regarding age and years of teaching experience, the sample covered all stages from young to senior teachers, with the majority being in the 31–40 age group and having 6–15 years of teaching experience. These teachers represent the backbone of frontline teaching and have a deep understanding of the impact and transformation brought by educational technology. In terms of educational background, all teachers held at least a bachelor’s degree, with 27.0% holding a master’s degree or above, reflecting the high educational attainment of the current teaching workforce. Additionally, the sample was relatively balanced across primary, junior high, and high school levels, and was predominantly composed of public school teachers (86.0%), which is consistent with the overall structure of basic education in China. Overall, the sample is diverse and broadly representative, providing reliable data support for the subsequent empirical analysis in this study.

## 4 SEM analysis

### 4.1 Reliability and validity testing of the scale

To ensure the scientific rigor of the measurement tools and the reliability of the data, this study conducted a systematic reliability and validity assessment of the questionnaire scale. First, the quality of the measurement model was examined, specifically evaluating the reliability and validity of each latent variable in the questionnaire. Reliability reflects the consistency and dependability of the measurement results, while validity ensures that the measurement tool accurately captures the theoretical constructs it is intended to measure. In this study, SPSS 26.0 and AMOS 24.0 software were used for data analysis. For reliability, Cronbach’s alpha and Composite Reliability (CR) were used as evaluation indicators; for validity, the Average Variance Extracted (AVE) was used to assess convergent validity. The results showed that the CR values for each dimension ranged from 0.639 to 0.719, all above the 0.60 threshold and close to or exceeding the recommended standard of 0.70, indicating good convergent validity for the measurement items of each latent variable. The AVE values for each dimension ranged from 0.841 to 0.885, well above the standard of 0.50, indicating that each latent variable could explain most of the variance in its measurement items and thus possessed good convergent validity.

In summary, the questionnaire scale achieved high academic standards in terms of both reliability and validity, providing a solid data foundation for subsequent SEM analysis and empirical research. The specific results of the reliability and convergent validity tests are shown in Table 3.

**Table 3:** Reliability and convergent validity test results of each latent variable.

Construct	Cronbach’s α	CR	AVE
TDA	0.839	0.639	0.841
TSC	0.880	0.710	0.880
PCE	0.845	0.701	0.876
PJC	0.868	0.687	0.868
WE	0.868	0.719	0.885
EA	0.874	0.700	0.875
PW	0.883	0.715	0.883

TDA, teaching design autonomy; TSC, teacher-student connection; PCE, professional competence expansion; PJC, proactive job crafting; WE, work engagement; EA, educational anxiety; PW, professional well-being.

To further verify the suitability of the data for factor analysis, this study conducted the KMO test and Bartlett’s test of sphericity on the sample data. The results showed that the KMO value was 0.884, which is well above 0.80, indicating that the sample data are highly suitable for factor analysis. The chi-square value for Bartlett’s test of sphericity was 4467.682, with 210 degrees of freedom and a significance level of *p* < 0.001, suggesting that the correlation matrix is not an identity matrix and that there are strong correlations among the variables. Therefore, the data meet the prerequisites for subsequent factor analysis. The specific test results are shown in Table 4.

**Table 4:** KMO and Bartlett’s test.

KMO		0.884
Bartlett’s sphericity	χ^2^	4467.682
	*df*-value	210
	*p*-value	0.000

As shown in Table 5, according to the Fornell-Larcker criterion for discriminant validity, the square roots of the AVE values for each latent variable are all located on the diagonal and are greater than the correlation coefficients between that variable and any other variable. This indicates that the latent variables exhibit good discriminant validity and can effectively distinguish between different theoretical constructs. Furthermore, Table 6 presents the discriminant validity test results based on the HTMT (Heterotrait-Monotrait Ratio) method. The HTMT values between all variables range from 0.222 to 0.540, which are significantly lower than the recommended threshold of 0.90, further confirming the discriminant validity among the latent variables.

**Table 5:** Discriminant validity (Fornell-Larcker criterion).

Variables	PW	EA	PJC	PCE	TDA	TSC	WE
PW	0.735						
EA	−0.390	0.792					
PJC	0.267	−0.276	0.624				
PCE	0.340	−0.339	0.247	0.637			
TDA	0.424	−0.407	0.232	0.395	0.842		
TSC	0.331	−0.311	0.150	0.276	0.329	0.733	
WE	0.320	−0.334	0.300	0.354	0.404	0.358	0.857

1. The diagonal elements represent the square root of the AVE value for each variable. 2. The off-diagonal elements represent the correlations between constructs. TDA, teaching design autonomy; TSC, teacher-student connection; PCE, professional competence expansion; PJC, proactive job crafting; WE, work engagement; EA, educational anxiety; PW, professional well-being.

**Table 6:** Discriminant validity (HTMT assessment criterion).

Variables	PW	EA	PJC	PCE	TDA	TSC	WE
PW							
EA	−0.512						
PJC	0.395	−0.393					
PCE	0.497	−0.478	0.392				
TDA	0.540	−0.498	0.320	0.540			
TSC	0.451	−0.408	0.222	0.403	0.419		
WE	0.403	−0.405	0.410	0.479	0.476	0.451	

TDA, teaching design autonomy; TSC, teacher-student connection; PCE, professional competence expansion; PJC, proactive job crafting; WE, work engagement; EA, educational anxiety; PW, professional well-being.

In summary, combining the Fornell-Larcker criterion and the HTMT assessment results, it can be concluded that the scale used in this study meets the academic requirements for both discriminant validity and convergent validity, providing a solid measurement foundation for subsequent **SEM** analysis.

### 4.2 Confirmatory factor analysis

As shown in Table 7, we conducted a systematic evaluation of the fit indices for the confirmatory factor analysis (CFA) model. Drawing primarily on the criteria proposed by Hu and Bentler in 1990 (49), we adopted a stringent two-index strategy (e.g., CFI ≥ 0.95) to obtain more reliable results. The findings indicate that the absolute, incremental, and parsimony fit indices all meet relevant academic standards. For absolute fit, CMIN/DF is 1.054 (<3); GFI and AGFI are 0.964 and 0.948 (> 0.90), respectively; and RMSEA is 0.012 (<0.05), indicating excellent model fit. Regarding incremental fit, the values of NFI, TLI, IFI, CFI, and RFI are 0.966, 0.998, 0.998, 0.998, and 0.957, respectively, all substantially exceeding the stricter recommended threshold of 0.95, further demonstrating superior model fit. For parsimony fit, PCFI, PNFI, and PGFI are 0.783, 0.758, and 0.676, respectively, each surpassing the recommended threshold of 0.50, suggesting a parsimonious and well-specified model structure.

**Table 7:** Validated factor analysis model fit.

Category	Fit index	Fit criterion	Model result	Model fit status
Absolute fit indices	CMIN/DF	<3	1.054	Yes
	GFI	>0.9	0.964	Yes
	AGFI	>0.9	0.948	Yes
	RMSEA	≤0.05	0.012	Yes
Incremental fit indices	NFI	>0.95	0.966	Yes
	TLI	>0.95	0.998	Yes
	IFI	>0.95	0.998	Yes
	CFI	>0.95	0.998	Yes
	RFI	>0.95	0.957	Yes
Parsimonious fit indices	PCFI	≥0.5	0.783	Yes
	PNFI	≥0.5	0.758	Yes
	PGFI	≥0.5	0.676	Yes

In sum, all fit indices meet or exceed recommended benchmarks, indicating that the CFA model exhibits good fit and providing a solid foundation for subsequent SEM analyses.

### 4.3 Path coefficients and hypothesis testing results

To test the theoretical hypotheses proposed in this study, we estimated and examined the significance of the path coefficients in the SEM. The key indicators, including the model path coefficients, standard errors (S.E.), critical ratios (C.R.), and significance levels (*p* values), calculated using Amos 24.0 software, are presented in Table 8.

**Table 8:** Model measurements.

Hypothesis				Estimate	S.E.	C.R.	*p*	Testing the hypothesis
H1	PW	←	TDA	0.238	0.066	3.618	0.000^***^	Established
H2	PW	←	TSC	0.187	0.059	3.147	0.002^**^	Established
H3	PW	←	PCE	0.161	0.075	2.158	0.031	Established
H4	PW	←	PJC	0.164	0.063	2.606	0.009^**^	Established
H5a	EA	←	TDA	−0.266	0.071	−3.750	0.000^***^	Established
H5b	EA	←	TSC	−0.196	0.065	−3.021	0.003^**^	Established
H5c	EA	←	PCE	−0.222	0.082	−2.704	0.007^**^	Established
H5d	EA	←	PJC	−0.234	0.069	−3.405	0.000^***^	Established

***Indicates *p* < 0.001; **Indicates *p* < 0.01; TDA, teaching design autonomy; TSC, teacher-student connection; PCE, professional competence expansion; PJC, proactive job crafting; WE, work engagement; EA, educational anxiety; PW, professional well-being.

Direct effects on teachers’ professional well-being (PW): Teaching design autonomy (TDA), depth of teacher-student connection (TSC), perceived competence expansion (PCE), and proactive job crafting (PJC) all exert significant positive effects on teachers’ professional well-being. Specifically, their standardized path coefficients (*β*) are 0.238 (*p* < 0.001), 0.187 (*p* < 0.01), 0.161 (*p* < 0.05), and 0.164 (*p* < 0.01), respectively. This indicates that when teachers perceive greater autonomy in teaching, deeper connections with students, stronger professional growth, and more proactive job crafting, their professional well-being is significantly enhanced. This suggests that higher levels of educational anxiety significantly reduce teachers’ professional well-being. Therefore, hypotheses H1, H2, H3, and H4 are all supported.

**Figure 3:**
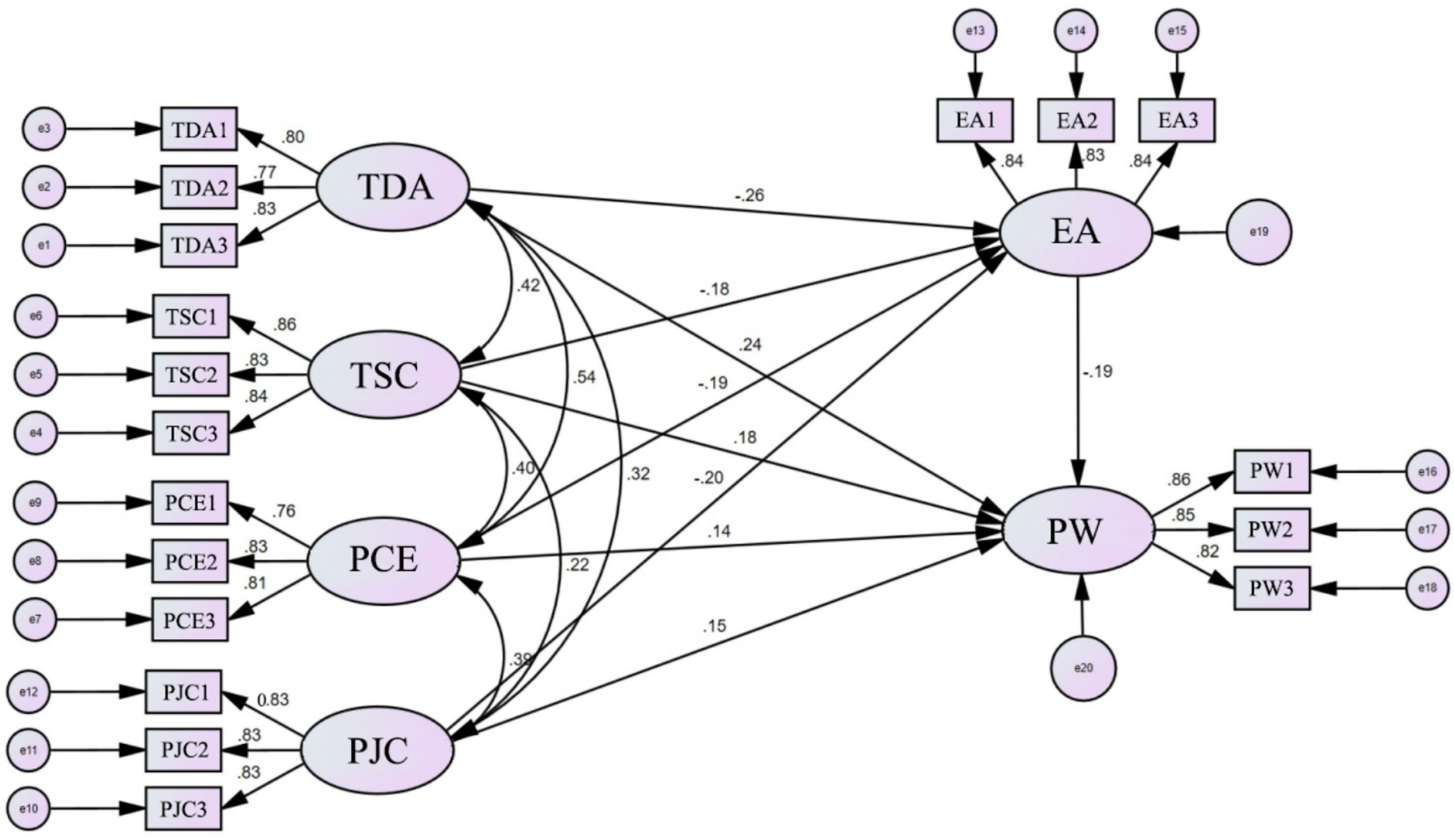
Path analysis results.

Direct effects on educational anxiety (EA): Teaching design autonomy (*β* = −0.266, *p* < 0.001), depth of teacher-student connection (*β* = −0.196, *p* < 0.01), perceived competence expansion (*β* = −0.222, *p* < 0.01), and proactive job crafting (*β* = −0.234, *p* < 0.001) all significantly and negatively predict educational anxiety. This result indicates that granting teachers more autonomy, helping them build positive teacher-student relationships, enhancing their sense of professional competence, and encouraging proactive job crafting are all important ways to effectively alleviate and reduce their educational anxiety. Therefore, hypotheses H6, H7, H8, and H9 are also supported (Figure 3).

### 4.4 Mediation effect analysis of educational anxiety

Table 9 presents the results of the mediating effect analysis of educational anxiety (EA) in the pathways between different independent variables and teachers’ professional well-being (PW). The results indicate that educational anxiety plays a significant partial mediating role between teaching design autonomy (TDA), depth of teacher-student connection (TSC), perceived competence expansion (PCE), proactive job crafting (PJC), and teachers’ professional well-being. Specifically, the indirect effect of TDA on OW through EA is 0.049 (95% confidence interval: 0.007 to 0.097, *p* = 0.019), the direct effect is 0.245 (*p* = 0.003), and the total effect is 0.294 (*p* = 0.001), all of which are statistically significant. Similarly, the indirect effects of TSC, PCE, and PJC on OW through EA are 0.034 (*p* = 0.023), 0.036 (*p* = 0.024), and 0.037 (*p* = 0.020), respectively, with their direct and total effects also being significant.

**Table 9:** Analysis of intermediation effects.

Mediation path	Effect	Estimate	Lower	Upper	*p*
TDA—EA—PW	Indirect Effect	0.049	0.007	0.097	0.019
	Direct Effect	0.245	0.089	0.404	0.003
	Total Effect	0.294	0.144	0.442	0.001
TSC—EA—PW	Indirect Effect	0.034	0.003	0.085	0.023
	Direct Effect	0.180	0.059	0.300	0.003
	Total Effect	0.214	0.103	0.327	0.001
PCE—EA—PW	Indirect Effect	0.036	0.003	0.090	0.024
	Direct Effect	0.145	0.007	0.290	0.045
	Total Effect	0.180	0.037	0.326	0.016
PJC—EA—PW	Indirect Effect	0.037	0.004	0.079	0.020
	Direct Effect	0.145	0.033	0.261	0.020
	Total Effect	0.183	0.075	0.292	0.005

TDA, teaching design autonomy; TSC, teacher-student connection; PCE, professional competence expansion; PJC, proactive job crafting; WE, work engagement; EA, educational anxiety; PW, professional well-being.

These results suggest that enhancing teachers’ teaching design autonomy, depth of teacher-student connection, perceived competence expansion, and proactive job crafting can not only directly improve teachers’ professional well-being, but also further enhance it by reducing educational anxiety. Educational anxiety thus serves as an important mediating bridge between these variables and teachers’ professional well-being.

### 4.5 Moderating effect of work engagement

Table 10 presents the analysis results of the moderating effect of teachers’ work engagement on the relationships between perceived competence expansion and professional well-being, as well as between proactive job crafting and professional well-being. The results show that the interaction term between work engagement and perceived competence expansion has a moderating effect of 0.099, with a standard error of 0.048, a *t*-value of 2.054, and a confidence interval of [0.041, 0.194], indicating that this moderating effect is significant. In other words, when the level of work engagement is high, the positive impact of perceived competence expansion on teachers’ professional well-being becomes more pronounced. Similarly, the interaction term between work engagement and proactive job crafting has a moderating effect of 0.118, with a standard error of 0.056, a T-value of 2.084, and a confidence interval of [0.007, 0.229], which is also statistically significant. This indicates that when teachers’ work engagement is high, the positive effect of proactive job crafting on professional well-being is further enhanced.

**Table 10:** Analysis of the moderating effect of teachers’ work engagement.

Hypothesis	Effect type	Effect size	Standard deviation	*t*-value	Confidence interval	
					LLCI	ULCI
H6	WE×PCE → PW	0.099	0.048	2.054	0.041	0.194
H7	WE×PJC → PW	0.118	0.056	2.084	0.007	0.229

PJC, proactive job crafting; WE, work engagement; PW, professional well-being.

**Figure 4:**
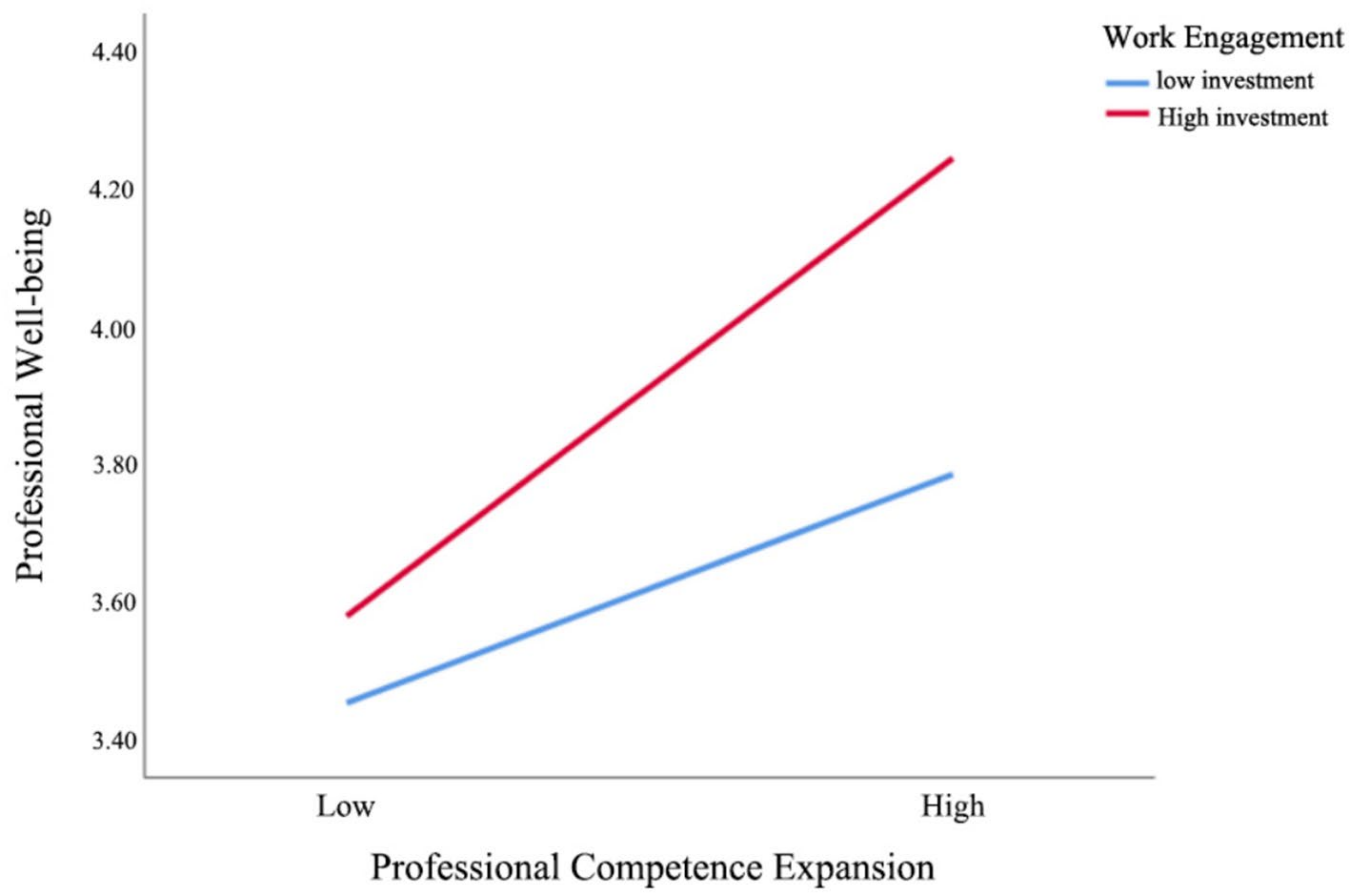
Moderating effect of teachers’ work engagement on the relationship between perceived competence expansion and professional well-being.

**Figure 5:**
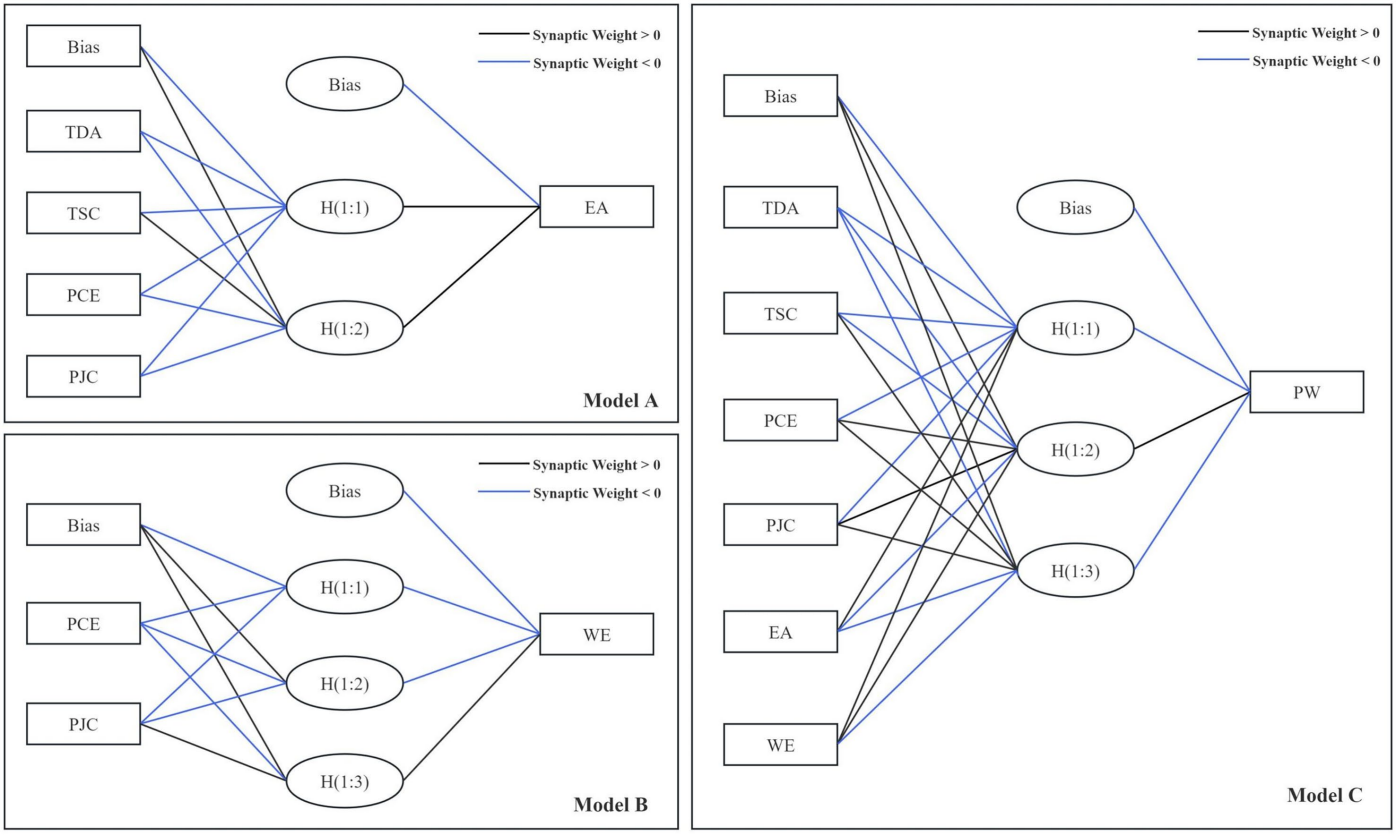
Moderating effect of teachers’ work engagement on the relationship between proactive job crafting and professional well-being.

In summary, teachers’ work engagement not only directly affects their professional well-being, but also strengthens the positive effects of perceived competence expansion and proactive job crafting on professional well-being, further revealing the key moderating role of work engagement in the formation mechanism of teachers’ professional well-being in the context of artificial intelligence technology (Figures 4, 5).

## 5 ANN neural network analysis

**Figure 6:**
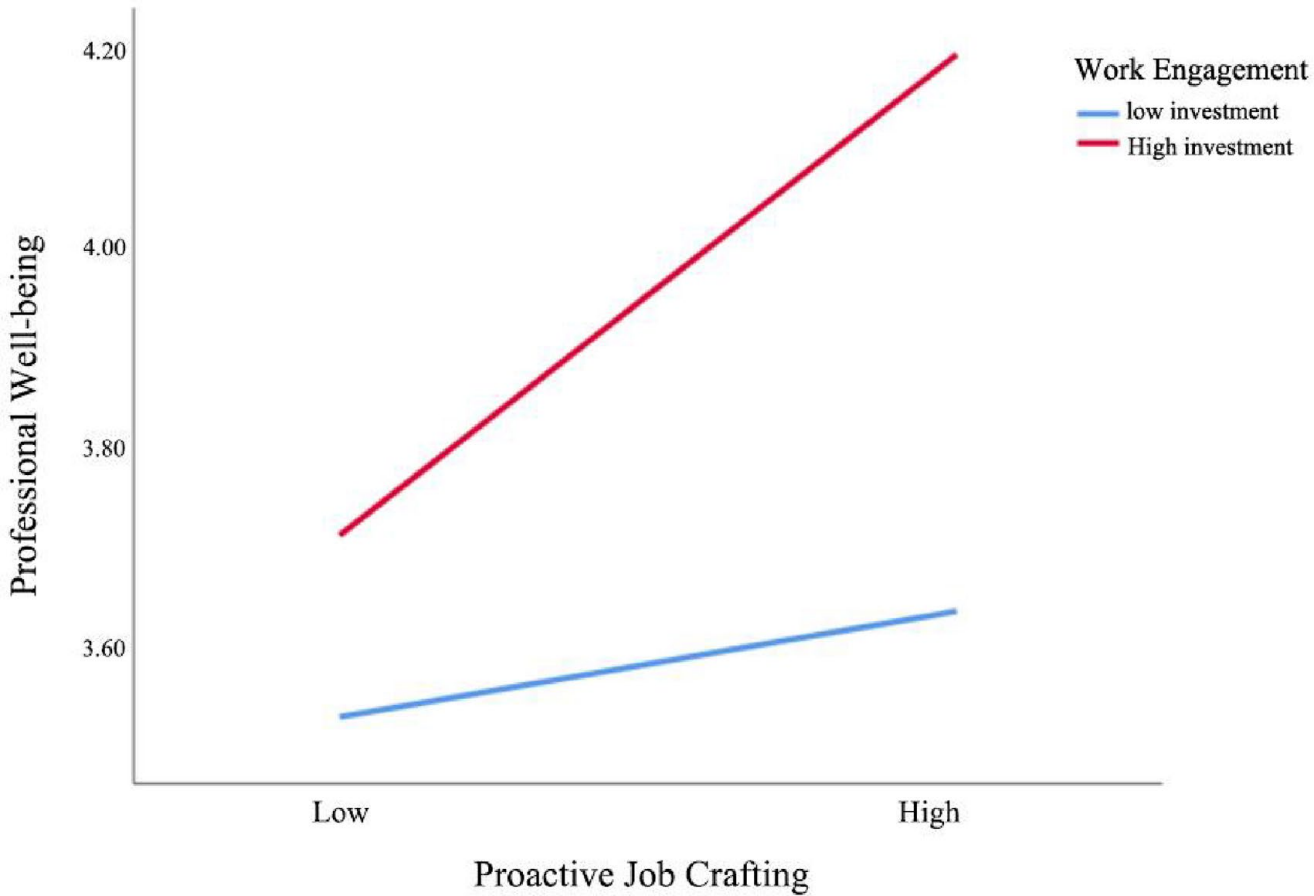
Artificial neural network model construction.

To overcome the limitations of traditional SEM, which primarily relies on linear relationship assumptions, and to further explore potential complex nonlinear relationships among variables, this study introduces the ANN method with the aim of constructing a hybrid SEM-ANN model for teacher well-being with higher predictive accuracy. This approach combines the theoretical validation strengths of SEM with the predictive capabilities of ANN, forming a complementary methodology. Drawing on the research paradigm of Liébana-Cabanillas et al. (50), this study adopts a two-stage analytical strategy: first, SEM is used to test and confirm the structural relationships of the theoretical model; then, the relationships validated by SEM serve as the foundational architecture for the neural network model, resulting in the construction of three hierarchical ANN models (A, B, and C), as shown in Figure 6.

Model A: Aims to predict the key mediating variable—educational anxiety (EA). Its input layer includes several antecedent variables that were validated in SEM as having significant effects on EA, namely Teaching Design Autonomy (TDA), Teacher-Student Connection (TSC), Professional Competence Expansion (PCE), and Proactive Job Crafting (TCA).

Model B: Aims to predict another key moderating variable—teachers’ work engagement (WE). Its input layer consists of Professional Competence Expansion (PCE) and Proactive Job Crafting (TCA).

Model C: Serves as the final predictive model, targeting the core dependent variable—teachers’ professional well-being (PW). The input layer of this model is the most comprehensive, incorporating not only the main external influencing factors (TDA, TSC, PCE, PJC), but also the output variables from Models A and B—educational anxiety (EA) and teachers’ work engagement (WE), thereby forming a complete predictive chain.

During the ANN modeling process, each model adopts a three-layer feedforward neural network structure, consisting of an input layer, a single hidden layer, and an output layer. The number of nodes in the hidden layer is determined based on the number of input variables and cross-validation results. The ReLU activation function is used in the hidden layer, while the output layer employs a linear activation function. The Adam optimizer is selected as the training algorithm with a learning rate of 0.001, and both early stopping and dropout techniques are applied to effectively prevent overfitting. All input variables are standardized prior to modeling. Through the integrated SEM-ANN approach, not only can the path relationships among variables be validated, but the complex nonlinear mechanisms between them can also be further revealed, providing a more scientific and precise theoretical basis and practical reference for enhancing teachers’ well-being.

### 5.1 Root mean square error validation

In this study, three multilayer perceptron neural network models (Models A, B, and C) were constructed, and a 10-fold cross-validation method was employed. The dataset was randomly divided into 10 groups, with 9 groups used as the training set and 1 group as the test set in turn, resulting in 10 independent rounds of training and testing. This approach effectively avoids the randomness caused by a single data split, making the model evaluation results more robust.

As shown in Table 11, Model A uses teaching design autonomy (TDA), depth of teacher-student connection (TSC), perceived competence expansion (PCE), and proactive job crafting (PJC) as input variables, with educational anxiety (EA) as the output variable. The average RMSE for the training and test sets are 0.357 and 0.350, respectively, with standard deviations of 0.152 and 0.319, indicating that the model has good fit and certain stability in predicting educational anxiety. Model B uses perceived competence expansion (PCE) and proactive job crafting (PJC) as inputs and work engagement (WE) as the output. The average RMSE for the training and test sets are 0.394 and 0.371, with standard deviations of 0.082 and 0.294, demonstrating that this model also exhibits high accuracy in predicting work engagement. Model C uses TDA, TSC, PCE, PJC, and EA as inputs, with teachers’ professional well-being (PW) as the output. The average RMSE for the training and test sets are 0.331 and 0.233, with standard deviations of 0.147 and 0.283. The test set RMSE for this model is the lowest, indicating that when multiple input variables are considered comprehensively, the model’s predictive ability for teachers’ professional well-being is the most superior.

**Table 11:** Root mean square error test for artificial neural network models.

Neural network	Model A		Model B		Model C	
	Input: TDA, TSC, PCE, PJC		Input: PCE, PJC		Input: TDA, TSC, PCE, PJC, EA	
	Ouput: EA		Ouput: WE		Ouput: PW	
	Training	Testing	Training	Testing	Training	Testing
ANN1	0.375	0.227	0.391	0.370	0.316	0.274
ANN2	0.341	0.490	0.403	0.340	0.317	0.353
ANN3	0.351	0.503	0.392	0.313	0.333	0.232
ANN4	0.328	0.327	0.396	0.487	0.301	0.128
ANN5	0.400	0.352	0.398	0.214	0.350	0.322
ANN6	0.376	0.321	0.406	0.523	0.347	0.142
ANN7	0.373	0.465	0.385	0.371	0.297	0.139
ANN8	0.334	0.287	0.394	0.355	0.358	0.257
ANN9	0.350	0.235	0.389	0.336	0.336	0.189
ANN10	0.339	0.295	0.388	0.397	0.350	0.291
Mean	0.357	0.350	0.394	0.371	0.331	0.233
SD	0.152	0.319	0.082	0.294	0.147	0.283

TDA, teaching design autonomy; TSC, teacher-student connection; PCE, professional competence expansion; PJC, proactive job crafting; WE, work engagement; EA, educational anxiety; PW, professional well-being.

This study uses root mean square error (RMSE) as the core evaluation metric for model performance; the smaller the RMSE value, the higher the predictive accuracy of the model. The training and test RMSE values for all three models are at relatively low levels, indicating that the ANN models perform well and are highly reliable in predicting and fitting the relationships among the variables in this study, thus providing strong technical support for further exploration of complex nonlinear relationships among variables.

### 5.2 Sensitivity analysis

Table 12 presents the results of the normalized relative importance analysis of different input variables on each output variable in the ANN models. By conducting multiple training sessions for the three models (A, B, and C), the average relative importance and normalized percentage of each input variable for the output variable were calculated.

**Table 12:** Analysis of the importance of normalization in artificial neural network models.

Neural network	Model A (Output: EA)				Model B (Output: WE)		Model C (Output: PW)					
	TDA	TSC	PCE	PJC	PCE	PJC	TDA	TSC	PCE	PJC	EA	WE
ANN1	0.362	0.122	0.200	0.315	0.481	0.519	0.171	0.150	0.237	0.166	0.166	0.110
ANN2	0.288	0.307	0.162	0.243	0.583	0.417	0.244	0.219	0.144	0.145	0.126	0.123
ANN3	0.258	0.192	0.286	0.264	0.553	0.447	0.109	0.087	0.222	0.231	0.272	0.078
ANN4	0.227	0.297	0.199	0.277	0.515	0.485	0.125	0.185	0.243	0.153	0.159	0.135
ANN5	0.466	0.421	0.049	0.064	0.561	0.439	0.281	0.173	0.038	0.210	0.144	0.154
ANN6	0.343	0.241	0.343	0.073	0.504	0.496	0.381	0.115	0.162	0.129	0.103	0.110
ANN7	0.326	0.353	0.223	0.098	0.558	0.442	0.166	0.167	0.213	0.171	0.148	0.135
ANN8	0.307	0.196	0.222	0.274	0.468	0.532	0.083	0.056	0.251	0.063	0.228	0.319
ANN9	0.229	0.238	0.267	0.267	0.540	0.460	0.178	0.153	0.192	0.158	0.203	0.116
ANN10	0.275	0.280	0.208	0.237	0.551	0.449	0.228	0.159	0.205	0.119	0.251	0.038
Average relative imporance	0.308	0.265	0.216	0.211	0.531	0.469	0.197	0.146	0.191	0.155	0.180	0.132
Normanlized relative importance (%)	100.00	86.04	70.13	68.51	100.00	88.32	100.00	74.11	96.95	78.68	91.37	67.01

TDA, teaching design autonomy; TSC, teacher-student connection; PCE, professional competence expansion; PJC, proactive job crafting; WE, work engagement; EA, educational anxiety; PW, professional well-being.

In Model A (output: EA), teaching design autonomy (TDA) has the highest normalized relative importance (100.000%), followed by depth of teacher-student connection (TSC, 86.039%), perceived competence expansion (PCE, 70.130%), and proactive job crafting (PJC, 68.507%). This indicates that teaching design autonomy is the most critical influencing factor in predicting educational anxiety. In Model B (output: WE), perceived competence expansion (PCE, 100.000%) is the most important, followed by proactive job crafting (PJC, 88.324%), demonstrating that perceived competence expansion plays a central role in enhancing teachers’ work engagement. In Model C (output: PW), teaching design autonomy (TDA, 100.000%) remains the most important predictor, followed by perceived competence expansion (PCE, 96.954%), educational anxiety (EA, 91.371%), proactive job crafting (PJC, 78.680%), depth of teacher-student connection (TSC, 74.112%), and work engagement (WE, 67.005%). This suggests that the improvement of teachers’ professional well-being depends not only on teaching design autonomy and perceived competence expansion, but is also jointly influenced by educational anxiety, proactive job crafting, teacher-student connection, and work engagement.

Overall, the results of the normalized importance analysis further reveal the core roles of each variable in different models, providing more intuitive and quantitative evidence for understanding the mechanisms influencing teachers’ professional well-being, work engagement, and educational anxiety. This also offers targeted references for subsequent educational management practices and policy formulation.

## 6 Conclusion and recommendations

### 6.1 Research findings

This study employed both SEM and ANN methods to thoroughly investigate the various factors influencing teachers’ professional well-being and their underlying mechanisms. The main findings are summarized in the following four aspects:

1. Direct effects of various factors on teachers’ professional well-being: Teaching design autonomy (TDA), depth of teacher-student connection (TSC), perceived competence expansion (PCE), and proactive job crafting (PJC) all have significant and direct positive effects on teachers’ professional well-being. This indicates that the greater the teaching autonomy, the stronger the teacher-student relationship, the higher the sense of professional growth, and the more proactive the job crafting, the higher the teachers’ professional well-being. Educational anxiety (EA) has a significant direct negative effect on teachers’ professional well-being. The higher the level of educational anxiety, the lower the teachers’ well-being. All direct effect hypotheses proposed in this study (H1 to H4) are supported by the data.
2. The mediating role of educational anxiety: Teaching design autonomy (TDA), depth of teacher-student connection (TSC), perceived competence expansion (PCE), and proactive job crafting (PJC) can all significantly and negatively predict educational anxiety. This suggests that enhancing these four aspects is an effective way to alleviate teachers’ educational anxiety. Educational anxiety thus plays a significant partial mediating role between the four positive factors—teaching design autonomy, depth of teacher-student connection, perceived competence expansion, and proactive job crafting—and teachers’ professional well-being. These four positive factors not only directly enhance teachers’ professional well-being, but also indirectly improve it by reducing educational anxiety.
3. The moderating effect of work engagement: Teachers’ work engagement (WE) plays a significant moderating role. Among teachers with high work engagement, the positive effect of perceived competence expansion on professional well-being is stronger, as is the positive effect of proactive job crafting on professional well-being. This indicates that work engagement can amplify the well-being benefits brought by professional growth and proactive job crafting.
4. Supplementary findings from the ANN models: The three constructed ANN models (predicting educational anxiety, work engagement, and professional well-being, respectively) all demonstrated low prediction errors (RMSE) and good stability, confirming the models’ effectiveness and reliability. Among all factors influencing educational anxiety, teaching design autonomy (TDA) is the most important. For work engagement, perceived competence expansion (PCE) is the most important factor. In the comprehensive model predicting teachers’ professional well-being, teaching design autonomy (TDA) remains the most important, followed by perceived competence expansion (PCE) and educational anxiety (EA). The importance rankings of influencing factors in the ANN and SEM models are relatively consistent.

## 7 Discussion

The core finding of this study—that work resources such as teaching design autonomy and perceived competence expansion play a key role in promoting teachers’ professional well-being—is highly consistent with the theoretical predictions of the mainstream Job Demands-Resources (JD-R) Model, and provides new empirical evidence for this theory in the current educational context. The JD-R model posits that job resources (such as autonomy, social support, and development opportunities) are critical drivers for enhancing employee work engagement and well-being, while also buffering the negative effects of job demands (51). The teaching design autonomy (TDA) and perceived competence expansion (PCE) identified in this study are typical structural and organizational resources within the JD-R model. Recent meta-analyses have confirmed that teacher autonomy is one of the most stable predictors of job satisfaction and reduced turnover intention (52). Notably, this study, through the ANN model, quantitatively highlights the primary importance of teaching design autonomy (TDA) among all predictive variables, further emphasizing that safeguarding teachers’ professional autonomy is not only a theoretical expectation but also the “first lever” in practice for enhancing teacher well-being. This finding also echoes research on the sources of “meaningfulness” in professional work, suggesting that when individuals can apply their expertise and judgment to core tasks, their sense of work meaning and well-being is maximized (53).

Another profound insight of this study lies in revealing the core mediating role of educational anxiety (EA) in the “resource–well-being” pathway. The findings show that positive work resources not only directly enhance well-being, but also indirectly protect and promote well-being by significantly reducing educational anxiety. This “dual-pathway” mechanism aligns with the logic of conservation of resources theory (54), which posits that individuals strive to acquire and protect valued resources, and that stress arises from the actual or potential loss of these resources. In this study, educational anxiety can be viewed as a state of psychological resource depletion caused by coping with high external demands. Work resources such as teaching autonomy and teacher-student connection constitute effective “resource reserves” that help teachers resist external pressures and prevent falling into a “loss spiral,” thereby maintaining psychological balance and well-being. Research by Carlijn and colleagues also found that strong school support systems can effectively buffer the stress and anxiety teachers experience when dealing with student behavioral problems (55). Therefore, the findings of this study provide important implications for teacher mental health interventions: simply offering “gain-oriented” benefits or incentives may have limited effects, while building resource-based support systems that effectively alleviate and prevent anxiety may be more fundamental and effective.

In addition, by verifying the moderating effect of work engagement (WE), this study elegantly elucidates the critical role of teachers’ individual agency in the process of resource transformation. The results indicate that work engagement can significantly amplify the positive effects of perceived competence expansion (PCE) and proactive job crafting (PJC) on well-being. This suggests that work resources do not have the same effect on everyone; teachers’ own psychological states act as a “catalyst” for the efficient transformation of resources into well-being. Teachers with high work engagement are more energetic, dedicated, and focused, and are more likely to actively seek and utilize development opportunities, as well as proactively reshape their work content and interpersonal boundaries to create a better work experience (56). This finding supports the role of work engagement and psychological capital as gain amplifiers in the JD-R model (57). Therefore, strategies to promote teacher well-being should be “two-pronged”: on the one hand, schools and educational systems need to provide abundant external work resources (58); on the other hand, it is equally important to stimulate and sustain teachers’ intrinsic work engagement through leadership and organizational culture, thereby building a virtuous cycle of “resource provision–individual engagement–well-being enhancement (59).”

## 8 Conclusions and implications

### 8.1 Conclusion

This study systematically explored the multiple factors influencing teachers’ professional well-being and their complex interaction mechanisms through a hybrid approach combining SEM and ANNs. The main conclusions are as follows: First, work resources such as teaching design autonomy, depth of teacher-student connection, perceived competence expansion, and proactive job crafting are significantly and positively associated with teachers’ professional well-being, while educational anxiety shows a significant negative association. Second, educational anxiety plays an important mediating role between these work resources and professional well-being, suggesting that the positive effects of these resources on well-being may be partially realized through the alleviation of teachers’ anxiety. Finally, teachers’ own level of work engagement appears to moderate the relationships between perceived competence expansion, proactive job crafting, and well-being, meaning that a high level of engagement may amplify the positive experiences brought by these two resources. The ANN model analysis further highlights the core importance of teaching design autonomy and perceived competence expansion in predicting teacher well-being and educational anxiety.

Based on these findings, this study provides several exploratory practical implications for enhancing teachers’ professional well-being. Given the strong association between teaching design autonomy, well-being, and anxiety levels, education administrators and policymakers might consider preserving and creating more professional autonomy for teachers within curriculum standards and teaching evaluation frameworks. This could mean reducing unnecessary administrative interventions and encouraging teachers to innovate in teaching based on student needs, thereby allowing teachers’ professional value to be more fully realized. At the same time, the importance of perceived competence expansion suggests that providing teachers with high-quality, systematic professional development opportunities may be an effective support direction. This should not only include skills training, but also focus on in-depth development programs that can stimulate teachers’ intrinsic motivation for growth and bring a sense of competence and achievement.

In addition, the results of this study offer potential ideas for supporting teachers’ mental health. As educational anxiety is a key mediating variable, its management and alleviation may be an important entry point for enhancing teacher well-being. At the school level, more comprehensive peer support systems and mentoring programs could be established to promote positive teacher-student relationships and buffer the anxiety caused by external pressures. For individual teachers, proactively engaging in job crafting and maintaining a high level of work engagement seem to be key to enhancing personal resources and effectively internalizing external support into well-being experiences. Therefore, guiding teachers to understand and practice job crafting strategies, as well as creating an organizational climate that fosters work engagement, are also worthy of attention and exploration in future educational management practices. It should be emphasized that this study is cross-sectional in design, and its conclusions mainly reveal associations rather than definitive causal relationships among variables. Therefore, the above practical implications should be regarded as hypothetical directions based on current data, and their effectiveness awaits further verification through future longitudinal or experimental intervention studies.

### 8.2 Research limitations and future directions

There are several important limitations in this study that should be considered when interpreting the results. First, the cross-sectional research design is the main limitation, as it prevents us from determining the causal relationships or temporal order among variables, and the observed associations may be subject to reverse causality or common causes. Future research should adopt longitudinal tracking designs or randomized controlled experiments to more accurately establish causal links among variables. Second, this study focused on specific work resources and psychological variables; future research could include more potential influencing factors, such as school organizational culture, leadership style, compensation, and colleague relationships, to build a more comprehensive model of factors affecting teachers’ professional well-being. In addition, the sample background of this study was not described in detail, so the generalizability of the conclusions to different educational stages, regions, or cultural contexts remains to be tested. Third, all variables were measured through self-report, which may introduce common method bias; future research could combine objective indicators or multi-source data to enhance the reliability of the results. Moreover, future studies could employ more diverse methods, such as qualitative interviews or mixed-methods research, to deepen the understanding of the formation process of teachers’ professional well-being. Finally, with the rapid development of AI technology, the challenges and opportunities faced by teachers are constantly evolving, requiring ongoing research to track this dynamic process.

## Data Availability

The original contributions presented in the study are included in the article/supplementary material, further inquiries can be directed to the corresponding author.
